# Supplementary material for: Interactions Between Spermine-Derivatized Tentacle Porphyrins and The Human Telomeric DNA G-Quadruplex
Source: Int J Mol Sci. 2018 Nov 21;19(11):3686. doi: 10.3390/ijms19113686 (PMC6274827; doi:10.3390/ijms19113686)

Supporting Information

Interactions between spermine-derivatized tentacle porphyrins and the human telomeric DNA G-quadruplex

Navin C. Sabharwal [1,2], Jessica Chen [1,3], Joo Hyun (June) Lee [1,4], Chiara M. A. Gangemi [5], Alessandro D'Urso [5,*], and Liliya A. Yatsunyk [1,*]

[1] Department of Chemistry and Biochemistry, Swarthmore College, Swarthmore, PA 19081 USA; navin.sabharwal.424@gmail.com (N.C.S.); ymchen.017@gmail.com (J.C.); jlee2143@gmail.com (J.H.L.);
[2] Lerner College of Medicine, Cleveland Clinic, Cleveland, OH 44195 USA
[3] School of Dental Medicine, University of Pennsylvania, Philadelphia, PA 19104 USA
[4] College of Dentistry, New York University, New York, NY 10010 USA
[5] Department of Chemical Science, University of Catania, 95125 - Catania, Italy; gangemichiara@unict.it
* Correspondence: adurso@unict.it (A.D'U.); lyatsun1@swarthmore.edu (L.A.Y.); Tel.: +39- 095-738-5095(A.D'U.); +01-610-328-8558, (L.A.Y.)

TCPPSpm4 and ZnTCPPSpm4 do not Aggregate in 0-40 μM Concentration Range in 5K Buffer

To determine the extinction coefficient for ZnTCPPSpm4, the exact amount of solid porphyrin was weighted and dissolved in a known amount of water. This sample was diluted in a precise way and the absorbance of an array of samples of different concentrations was measured to determine the extinction coefficient of ZnTCPPSpm4 using the slope of the best fit line. The value of extinction coefficient at 424 nm is 1.34×10^5 $M^{-1}cm^{-1}$. Concentrated sample of TCPPSpm4 at precise concentration was prepared using previously determined extinction coefficient of 3.0×10^5 $M^{-1}cm^{-1}$ at 415 nm at pH of 6.5 (55). This sample was used to conduct dilution studies.

Figure S1. Beer's Law study of (**A**) TCPPSpm4 and (**B**) ZnTCPPSpm4 in 5K buffer, pH 7.2. A linear relationship between absorbance and concentration of porphyrin indicates no change in the aggregation state of the porphyrins up to 40 μM.

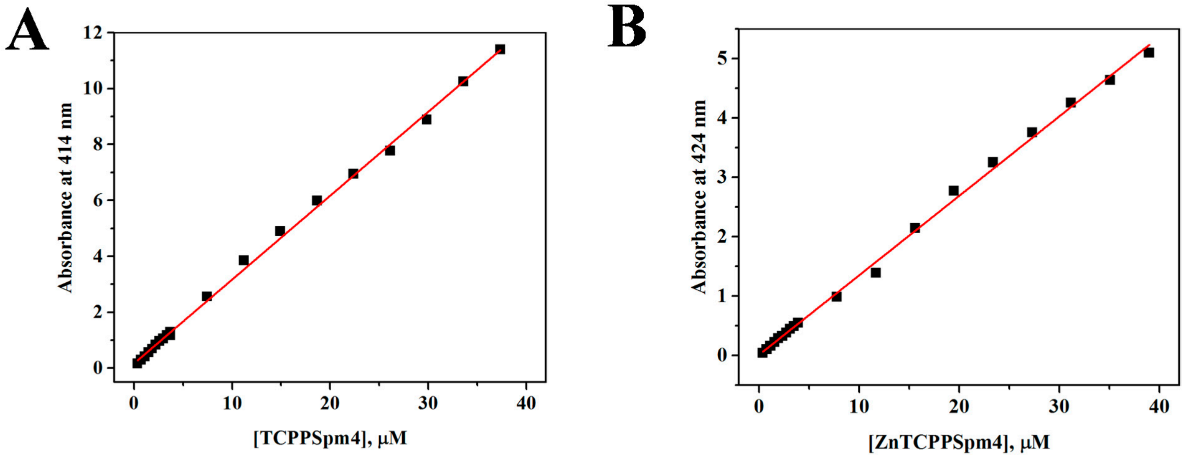

Fluorescent Titration of Porphyrins with Tel22 Leads to Dramatic Decrease in the Peak Intensity

Figure S2. Representative steady-state fluorescence emission spectra for (**A**) 0.33 µM TCPPSpm4 and (**B**) 0.47 µM ZnTCPPSpm4 upon titration with Tel22. Titration was stopped at 19.5 fold excess of Tel22 in the case of TCPPSpm4 and 9.1 fold excess of Tel22 in the case of ZnTCPPSpm4. Insets show the fluorescence spectra of poprhyrin-Tel22 complexes at saturating amount of Tel22. We attempted to extract binding constants from the titration data, but were not successful.

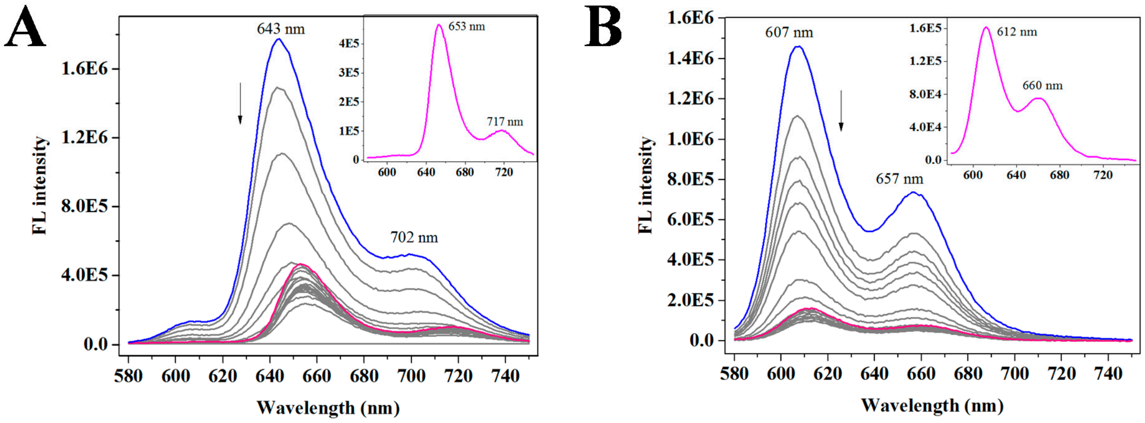

Figure S3. FRET stabilization and competition studies. Stabilization of 0.2 μM F21D in 5K buffer by (**A**) TCPPSpm4 and (**C**) ZnTCPPSpm4 in FRET melting assays. Melting of F21D alone is shown by black symbols. FRET competition studies using 0.2 μM F21D with (**B)** 0.75 μM TCPPSpm4 or (**D)** 2.2 μM ZnTCPPSpm4 in the presence of increasing amount of CT DNA. Equivalents of CT DNA relative to F21D are specified in the legend. Concentration of porphyrins was chosen such as to achieve similar starting Tm for the first sample before any CT DNA was added in order to facilitate the comparison. Control samples include i) F21D alone, and ii) F21D in the presence of 80 eq. CT DNA in the absence of porphyrins to test the effect of CT on F21D. Note, presence of CT does not change the Tm of F21D. Each experiment was run in duplicate and was repeated at least three times.

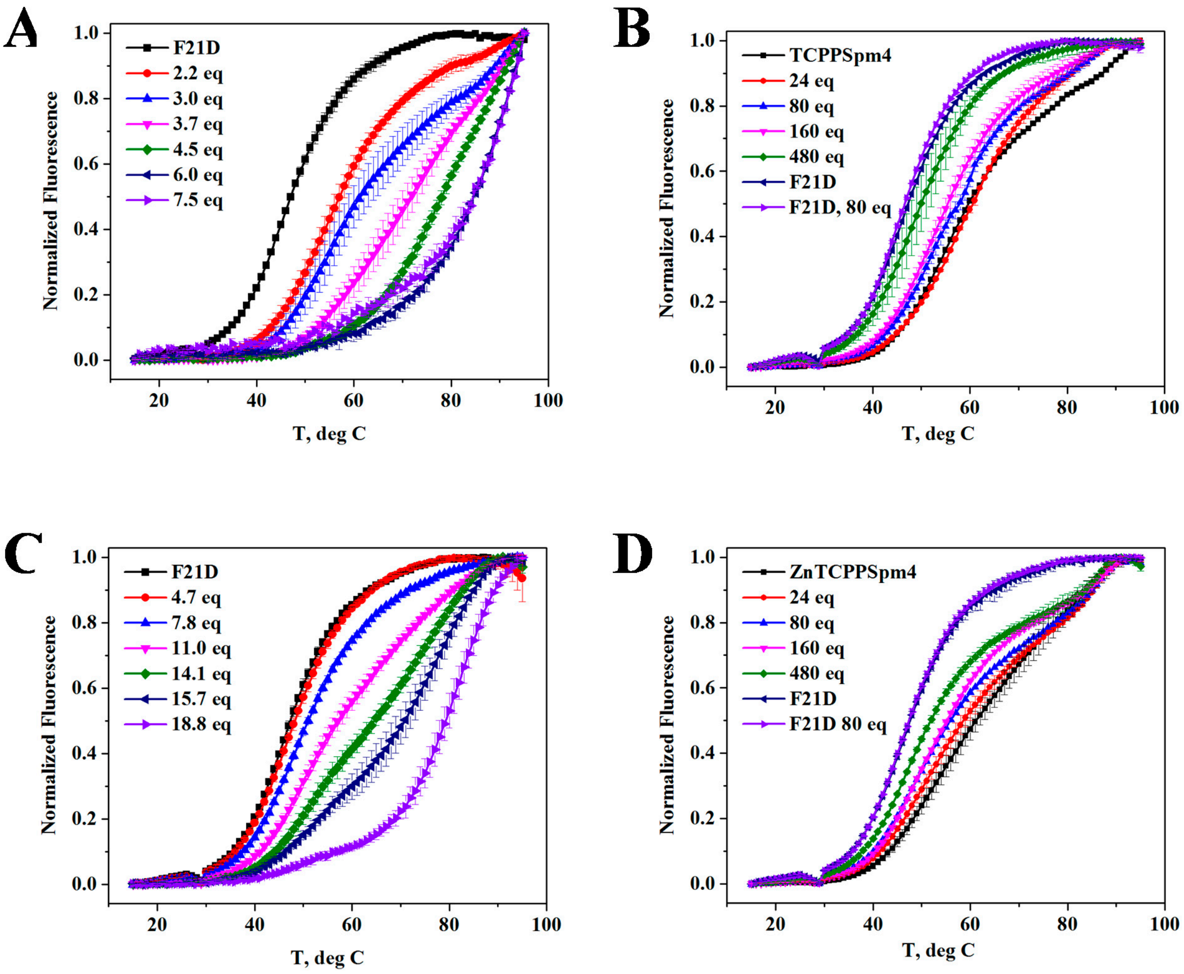

CD Melting Studies of Tel22 in the Presence of up to 2 eq. of Porphyrins Reveal no-to-Weak Stabilization

We performed CD melting experiments using Tel22 alone and Tel22 in the presence of 1 and 2 equivalents of porphyrins. In 5K buffer Tel22 has Tm of 50.2 ± 0.2 °C. Its stability increases only by 1-2 °C in the presence of 1-2 eq. of TCPPSpm4 and ZnTCPPSpm4 (associated error ± 1 °C). While the CD data seemingly contradict the results from FRET melting studies, one needs to keep in mind that in FRET efficient stabilization was only observed above 2 eq. of TCPPSpm4 and 8 eq. of ZnTCPPSpm4.

Figure S4. CD melting study for 5.4 μM Tel22 in the presence of 4.05 and 8.10 μM ZnTCPPSpm4. No DNA stabilization was observed in this study.

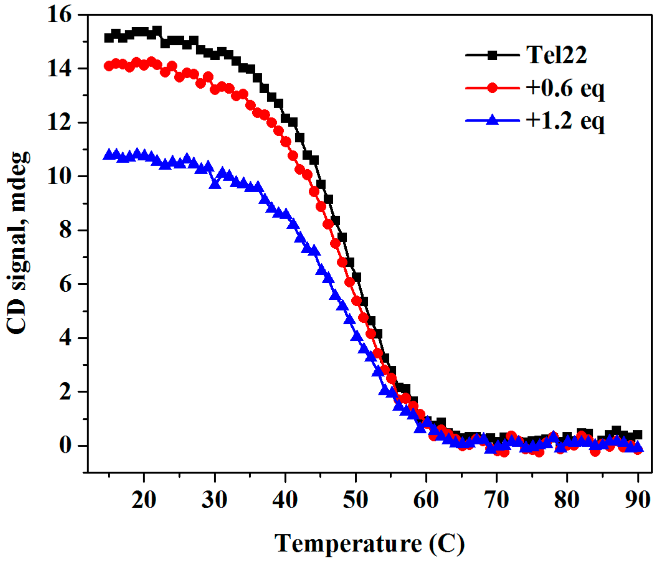

The Presence iCD Confirms Close Contacts between Porphyrins and Tel22 Aromatic Systems as well as the Stoichiometry Obtained in UV-vis

Figure S5. Induced CD signature of TCPPSpm4-Tel22 and ZnTCPPSpm4-Tel22 complexes. Representative CD titrations of (**A**) 1.6 µM TCPPSpm4 with of 97 µM Tel22 and (**B**) 2.2 µM ZnTCPPSpm4 with 97 µM Tel22 at 20 °C. The data in both titrations were smoothed using Savitzky–Golay smoothing filter with a 13-point quadratic function. Both data sets are plotted using the same y-scale for ease of comparison. Overlay of two titrations monitored at the specified wavelengths for (**C**) TCPPSpm4 and for (**D**) ZnTCPPSpm4 with Tel22 in the iCD region. Dashed lines denote the maxima/minima on the titration curves and correspond to the optimal stoichiometry for porphryin-Tel22 complexes obtained in UV-vis.

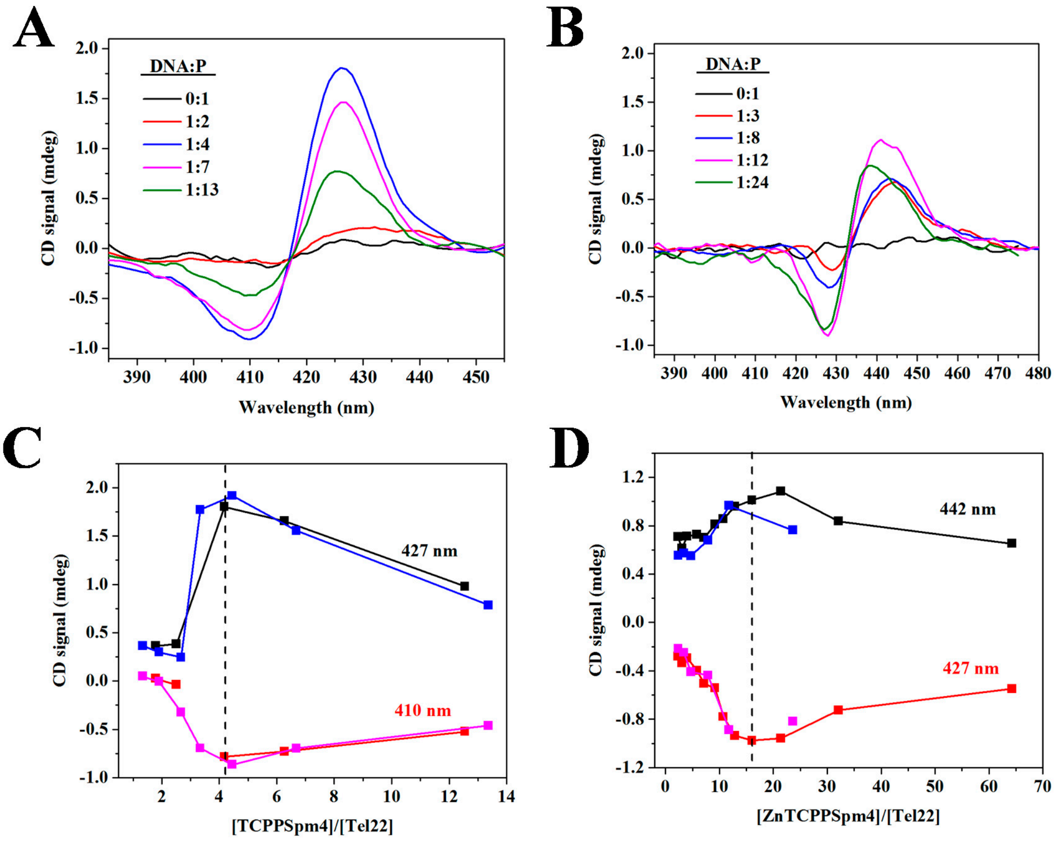

Supplement: Supplementary file 1 [file ijms-19-03686-s001.pdf]
